# Antifungal Activity of Chitosan Nanoparticles Encapsulated With *Cymbopogon martinii* Essential Oil on Plant Pathogenic Fungi *Fusarium graminearum*

**DOI:** 10.3389/fphar.2018.00610

**Published:** 2018-06-06

**Authors:** Naveen K. Kalagatur, Oriparambil S. Nirmal Ghosh, Naveen Sundararaj, Venkataramana Mudili

**Affiliations:** ^1^Food Microbiology Division, Defence Food Research Laboratory, Mysuru, India; ^2^Centre for Nanoscience and Technology, Pondicherry University, Puducherry, India; ^3^Microbiology Department, PSG College of Arts and Science, Coimbatore, India; ^4^Toxicology and Immunology Division, DRDO-BU Centre for Life Sciences, Bharathiar University, Coimbatore, India

**Keywords:** mycotoxins, *Fusarium graminearum*, deoxynivalenol, zearalenone, *Cymbopogon martinii* essential oil, chitosan encapsulated nanoparticles, reactive oxygen species, ergosterol

## Abstract

Application of synthetic fungicides in agricultural commodities has been restricted due to development of fungicide resistance fungi and deleterious impact on environment and health of farm animals and humans. Hence, there is an urge for development of mycobiocides, and the present study was undertaken to determine the antifungal activity of *Cymbopogon martinii* essential oil (CMEO) on post-harvest pathogen *Fusarium graminearum*. The CMEO was extracted by hydrodistillation and GC-MS chemical profile revealed the presence of 46 compounds and abundant was geraniol (19.06%). The minimum inhibitory concentration and minimum fungicidal concentration of CMEO were determined as 421.7 ± 27.14 and 618.3 ± 79.35 ppm, respectively. The scanning electron microscopic observation of CMEO exposed macroconidia was exhibited a detrimental morphology with vesicles, craters, protuberance, and rough surfaces related to control fungi. The CMEO induced the death of fungi through elevating intracellular reactive oxygen species and lipid peroxidation, and depleting ergosterol content. Regrettably, essential oils are highly volatile and become unstable and lose their biological features on exposure to light, heat, pH, moisture, and oxygen. To overcome these issues, chitosan encapsulated CMEO nanoparticles (Ce-CMEO-NPs) were prepared. The synthesized Ce-CMEO-NPs have spherical morphology with Zeta potential of 39.3–37.2 mV and their corresponding size was found in range of 455–480 nm. The Fourier transform infrared analysis confirmed that bio-active constituents of CMEO were well stabilized due to chitosan conjugation and successfully formed Ce-CMEO-NPs. The *in vitro* release assay observed that the release of CMEO is stabilized due to the complex formation with chitosan and thereby, increases the lifetime antifungal activity of CMEO by gradual release of antifungal constituents of Ce-CMEO-NPs. In conclusion, antifungal and antimycotoxin activities of CMEO and Ce-CMEO-NPs against *F. graminearum* were assessed in maize grains under laboratory conditions over a storage period of 28 days. Interestingly, Ce-CMEO-NPs were presented efficient and enhanced antifungal and antimycotoxin activities related to CMEO, and it could be due to perseverance of antifungal activity by controlled release of antifungal constituents from Ce-CMEO-NPs. The study concluded that Ce-CMEO-NPs could be highly appropriate as mycobiocides in safeguarding the agricultural commodities during storage period in agricultural and food industries.

## Introduction

*Fusarium graminearum* [telemorph Gibberella zeae (Schwein)] is a sporadic plant pathogen, causes Fusarium head blight (FHB) in barley and wheat, stalk rot and Gibberella ear rot in maize, and one of the utmost liable for economic setback in the agriculture and food industry worldwide (Dweba et al., 2017). *F. graminearum* infestation of agriculture commodities fetches the rancidity along with the loss in grain yield and quality, including texture, color, odor, and nutrition (Morgavi et al., 2007; Okeke et al., 2015). As well, *F. graminearum* is a prominent producer of hazardous Fusarium mycotoxins, including deoxynivalenol (DON), nivalenol (NIV), and zearalenone (ZEA), which were reported to cause deleterious health issues in the farm animals and humans, such as nephrotoxic, immunotoxic, neurotoxic, hepatotoxic, genotoxic, reproductive, and developmental defects (Morgavi et al., 2007; Venkataramana et al., 2014; Kalagatur et al., 2017). Besides, International Agency on Research and Cancer (IARC) has confirmed the carcinogenicity of Fusarium mycotoxins and classified into group 3 agents (IARC, 1999). Concerning these worries, the European Union (EU), Joint FAO/WHO Expert Committee on Food Additives (JECFA), and various nations have recommended stringent regulatory levels for Fusarium mycotoxins in agricultural and food products (JECFA, 2000; European Commission, 2007).

Regrettably, *F. graminearum* and its risky toxins are coherent contaminants of wide range of agricultural commodities worldwide (Richard et al., 2003; Pasquali et al., 2016; Tralamazza et al., 2016; Ok et al., 2018). Recently, Beccari et al. (2018) have detected the higher incidence of FHB in Durum wheat samples of central Italy and reported that 22% fungal species were *F. graminearum*. In Mediterranean countries of Europe, Gorczyca et al. (2018) have detected yield loss and contamination of mycotoxins in Durum wheat owed to FHB incidence. In the same way, Vogelgsang et al. (2017) in Switzerland haev surveyed the incidence of DON and ZEA between 2007 and 2014 in wheat and noticed an overall mean concentration of 607 μg/kg of DON and 39 μg/kg of ZEA. Intolerably, 11% and 7% of wheat samples were surpassed the EU regulatory limits of DON and ZEA, respectively, and found unfit for consumption. Similarly, Mallmann et al. (2017) during 2008–2015 in Brazil have measured the prevalence of FHB and detected 73% and 38% of wheat samples, 67% and 41% of barley samples were contaminated with DON and ZEA, respectively. Furthermore, Kos et al. (2017) in Serbia have noticed 260.1–1388, 260.4–9050, and 252.3–6280 μg/kg of DON in maize samples during 2013, 2014, and 2015, respectively. Likewise, Mudili et al. (2014) and Ramana et al. (2011) too enlightened the prevalence of *F. graminearum* in rice, finger millet, and maize grains originated from Southern region of India and perceived 72–94 μg/kg of DON attendance in freshly harvested maize grains.

The status quo apprehensions the insistent incidence of *F. graminearum* and its toxins in agricultural commodities, and demand effective approaches from microbiologists and food technologists to overcome these problems in agriculture and food industry. However, the application of synthetic fungicides has not been acceptable because of increasing concern on multiple drug resistance, environmental safety, and deleterious health issues in humans and animals. Over the last decade, plant-originated mycobiocides, exclusively essential oils have attained a considerable attention as of its non-toxic and eco-friendly (Hyldgaard et al., 2012; Kalagatur et al., 2015; George et al., 2016; Iram et al., 2016; Kumar et al., 2016; Vanhoutte et al., 2016; Muniyandi et al., 2017). In recent times, nanotechnology has provided great assistance to improve the microbiological safety of food and feed matrices. Particularly, nanomaterials of chitosan were found eco-friendly and safe, and their application in food is highly satisfactory by consumer, food industry, and regulatory agencies (Yeung et al., 2016; Niaz et al., 2017; Siddaiah et al., 2018). Furthermore, essential oils are volatile and lose their stability and bio-functional features on exposure to temperature, light, pH, moisture, and oxygen (Jamil et al., 2016). In this context, the encapsulation of essential oils with chitosan by nanotechnology approach was recognized as a finest approach to safeguard the stability and bio-functional features of essential oils (Barreto et al., 2016; Yeung et al., 2016).

In this context, essential oil obtained from leaves of *Cymbopogon martinii* was encapsulated with chitosan and used for reduction of growth and mycotoxins of *F. graminearum*. The *C. martinii* also known as Indian geranium, motia, and rosha, is a tropical herbaceous grass belong to family Poaceae and extensively distributed subtropical parts of Africa, America, and Asia (Duke, 1993). This plant is a native of India and grows as wild and irrigated crop in Western Ghats found among the states of Andhra Pradesh, Karnataka, Kerala, and Tamil Nadu, and in some parts of North Eastern India (Rao, 2001; Gupta et al., 2015). The aerial parts of plant such as leaf, stem, and inflorescence are considered as decent sources for essential oil and profoundly rich in bioactive chemical constituents like caryophyllene, geraniol, geranyl acetate, linalool, myrcene, limonene, humulene, selinenes, etc. (Rao et al., 2005; Cannon et al., 2013; Verma et al., 2013; Kakaraparthi et al., 2015). *C. martinii* essential oil (CMEO) is considered as non-toxic, non-sensitizing and non-irritant, and not reported for any deleterious health effects and recognized as “Generally Regarded as Safe” (GRAS) by the US Food and Drug Administration, and extensively used as antimicrobial, antihelmintic, cosmetic, toiletry, pharmaceutical, preservative, stress revealer, convalescence, insect, and mosquito repellents, and also as an ingredient in herbal tea, non-alcoholic beverages, flavor in tobacco products, traditional and baked foods, and all these features reflect the potency of CMEO in essential oil industry (Kumar et al., 2007; Tsai et al., 2011; Gupta et al., 2015).

In the present study, CMEO was extracted from leaves by hydrodistillation technique and chemical profile was determined by GC-MS analysis. The antifungal activity of CMEO on *F. graminearum* was determined in terms of minimum inhibitory concentration (MIC) and minimum fungicidal concentration (MFC) by micro-well dilution method, and further, it was confirmed by scanning electron microscopic (SEM) observation of macroconidia. The antifungal mechanism of CMEO was revealed by determining the content of intracellular reactive oxygen species (ROS), lipid peroxidation, and ergosterol. The chitosan encapsulated CMEO nanoparticles (Ce-CMEO-NPs) were prepared by emulsification technique and characterized by Fourier transform infrared (FT-IR), SEM, Zeta potential and size, and *in vitro* release of essential oil. Finally, *in vitro* antifungal and antimycotoxin activities of CMEO and Ce-CMEO-NPs on *F. graminearum* were comparatively evaluated in maize grains.

## Materials and Methods

### Chemicals and Materials

Czapek dox (CD) agar and broth, and peptone were obtained from HiMedia (Mumbai, India). Dichloro-dihydro-fluorescein diacetate (DCFH-DA), ergosterol, DON (99.99% pure), ZEA (99.99% pure), chitosan (high purity, 99% degree of deacetylation, and molecular weight of 100 kDa), and Whatman no. 1 qualitative filter papers were obtained from Sigma-Aldrich (Bengaluru, India). The immunoaffinity columns of DON and ZEA were obtained from Vicam (Waters, United States). Acetone, acetonitrile, anhydrous sodium sulfate, water, Tween 80, and other chemicals of fine grade were obtained from Merck Millipore (Bengaluru, India) and plasticware were from Eppendorf (Bengaluru, India). The mature, freshly harvested, and thoroughly dried maize grains were obtained from local agriculture market, Mysuru, India.

### Collection and Characterization of CMEO

#### Collection of Plant Material and Extraction of Essential Oil

The leaves of the *C. martinii* were collected prior inflorescence from Ooty, Nilgiris district, Tamil Nadu state, India, and voucher (PEO: 24) was safeguarded. The collection location was sited at latitude 11.41° N and longitude 76.70°E with an altitude of 7350 feet above the sea level. The plant material was cleaned with distilled water and dried in sheltered enclosure at room temperature for 2 weeks and crushed to fine powder. A quantity of 250 g dried powder was employed for extraction of essential oil by hydrodistillation using a Clevenger type device (Teknik, Ambala, India) implementing the technique of European Pharmacopeia (Council of Europe, 1997). The moisture content was detached from oil by aeration over anhydrous sodium sulfate and stored in sealed dark-brown glass vials at 4°C for further studies. The percentage yield of oil was calculated from the dry weight of the plant material by applying the following formula:

Yield of oil (%) = w(EO)/w(P) × 100

where *w* (EO) and *w* (P) were weight of essential oil and dried plant material, respectively.

#### GC-MS Analysis

The chemical constituent of CMEO was revealed using GC-MS (PerkinElmer Clarus 600 C, Waltham, United States) attached with DB-5MS silica fused column (30 m × 0.25 mm; 0.25 μm film thickness), flame-ionization detector (FID), and Turbo Mass software program. Briefly, CMEO was diluted with acetone (10 μL/mL) and 1 μL was injected in a split mode of 1:30. Helium (He) was carrier gas at a delivery rate of 1 mL/min. The mass spectra of compounds were determined at an *m*/*z* range of 40–450 and ionization energy of 70 eV under vacuum. Identification of the compounds was carried out matching the mass spectra and retention indices of n-alkanes (C-9 to C-24) with NIST05.LIB/Wiley 8th Edition and literature of Adams (2007), respectively. The quantification of compounds was attained from GC peak areas with devoid of FID response correction factors.

### Antifungal Activity of CMEO

Antifungal activity of CMEO was tested on mycotoxigenic *F. graminearum* (DFRL FgM: 18), which was originally isolated from freshly harvested maize kernels in our earlier study (Mudili et al., 2014). The fungal culture was allowed to grow for 14 days at 28°C on CD agar and spores were collected in sterile peptone water made up of 0.05% of Tween 80 by soft scrap, and the sum of spores was attained to 10^6^ spores/mL using a hemocytometer and used in further studies. The antifungal activity of CMEO was determined by the micro-well dilution method. Furthermore, deleterious effects of CMEO on micromorphology of macroconidia were observed by SEM. The mechanism of antifungal activity of CMEO was unveiled by comparing the content of intracellular ROS, lipid peroxidation, and ergosterol content of CMEO-treated fungi with untreated control fungi.

#### Micro-Well Dilution Method

The antifungal effect of CMEO on *F. graminearum* was determined in terms of MIC and MFC by the micro-well dilution method in 96-well microtiter plate adopting the methodology of Clinical and Laboratory Standards Institute [CLSI] (2008) with minor modifications. A volume of 10 μL spore suspension (1 × 10^6^ spores per mL) was added to wells of 96-well microtiter plate and blended with different concentrations of CMEO (up to 1000 ppm) and final volume was attuned to 100 μL with CD broth. The wells devoid of CMEO and with nystatin were control and reference, respectively. The plate was incubated for 3 days at 28°C. Following, MIC was noticed as the minimum concentration of CMEO at no visible fungal growth was observed. The incubation period was continued for 7 days at 28°C and 10 μL was spread on CD agar plates and allowed to grow for 3 days at 28°C and MFC was noticed as the minimum concentration of CMEO at no visible fungal growth was observed on CD agar plates.

#### Determination of Intracellular ROS

The antifungal mechanism of CMEO was evidenced by estimating the content of intracellular ROS in CMEO treated (up to MFC value of 618.3 ppm) and untreated control samples using DCFH-DA adopting a technique of Liu et al. (2010) with minor modification. A different concentration of CMEO (up to MFC value) was mixed with 10 μL spore suspension (1 × 10^6^ spores per mL) in 96-well microtiter plate and total volume brought up to 100 μL with CD broth and incubated for 7 days at 28°C. The sample not treated with CMEO was considered as a control. Subsequently, samples were stained for 20 min with 5 μM DCFH-DA. The fluorescence intensity was determined at excitation of 495 nm and emission of 550 nm in a multimode reader (Biotek-H1M synergy, Germany) and percentage of intracellular ROS in CMEO-treated samples was determined with respect to the untreated control (100%). The phase-contrast and fluorescent images were captured under an EVOS fluorescence microscope (Life Technologies, United States) at a magnification of 200 μm.

#### Determination of Lipid Peroxidation

The effect of CMEO on lipid peroxidation of fungi was done as per methodology of Gao et al. (2016) with minor modifications. Different concentrations of CMEO (up to MFC value of 618.3 ppm) along with 10 μl of fungal spore suspension were added to 100 mL of CD broth in 250 mL Erlenmeyer flask and incubated for 7 days at 28°C, and fungal sample without CMEO was considered as a control. Next, fungal mycelia were separated from broth by sieving through Whatman no. 1 filter paper and used for estimation of lipid peroxidation. The amount of malondialdehyde (MDA) present in the fungal sample was determined as an indicator of the level of lipid peroxidation. The quantification of MDA was done using spectrometric-dependent lipid-peroxidation assay kit as per instructions from the manufacturer (Sigma-Aldrich).

#### Determination of Ergosterol

The impact of CMEO on ergosterol content of *F. graminearum* was determined following the methodology of Silva et al. (2010) and Sellamani et al. (2016) with minor modifications. Different concentrations of CMEO (up to MFC value of 618.3 ppm) were exposed to 10 μL spore suspension (1 × 10^6^ spores per mL) in 100 mL CD broth of 250 mL Erlenmeyer flask and incubated for a period of 7 days at 28°C. Subsequently, the mycelia were separated from the broth employing Whatman no. 1 filter paper. A quantity of 5 mg of mycelia was used for determination of ergosterol and blended with 1 mL of ethanol, 5 mL of methanol, and 0.5 g of potassium hydroxide. The mixture was kept in dark at 70°C for 40 min and supernatant was collected by centrifugation at 8000 rpm for 10 min and thoroughly mixed for 10 min with n-hexane (v/v, 1:1). The blend was dried out at 60°C using water bath and re-solubilized in 1 mL of methanol and quantification of ergosterol was deducted from the calibration curve of standard ergosterol and results were compared with CMEO untreated control. A calibration curve for standard ergosterol was constructed with peak area vs. concentration of ergosterol using HPLC (Shimadzu, Kyoto, Japan). The system was equipped with C18 column, 5 μm thickness, 250 mm × 4.60 mm (Phenomenex, CA, United States) and working conditions were as follows: stock solution of ergosterol was prepared in methanol, injecting volume was 25 μL for both test and standard samples, UV detector was used with an excitation of 240 nm and emission of 300 nm wavelengths, and mobile phase was acetonitrile and water in a ratio of 6.5:3.5 (v/v) with a flow rate of 1 mL/min.

#### SEM Observation of Macroconidia

The SEM observation study was undertaken to know the effect of CMEO on the micro-morphological structure of macroconidia. The fungi were grown for 7 days on CD agar plates and 1 cm^2^ of mycelia mat was collected and inoculated on to CD agar slides that contain MIC and MFC concentrations of CMEO and incubated for 7 days at 28°C. The fungi not treated with CMEO were control. Next, mycelia were fixed on conducting dual side carbon tape and coated with gold using a sputter coater (Emitech, model no.: SC7620) and dried out in CO_2_ chamber. The micro-morphological structure of macroconidia was captured in an environmental mode under SEM (FEI, model no.: Quanta 200) at a magnification of 5000×.

### Preparation and Characterization of Ce-CMEO-NPs

#### Preparation

The preparation of Ce-CMEO-NPs with different concentrations of CMEO was done as per methodology of Ribeiro et al. (2013). Briefly, different ppm of CMEO (up to 1000 ppm) and Tween 80 at a ratio of 2:1 (v/v) were added to 100 mL of 4% chitosan solution and homogenized continuously for 10 min at 50 rpm under a magnetic stirrer. Next, attained emulsion was incubated at 28 ± 2°C for 3 days to form Ce-CMEO-NPs. The characterizations of prepared Ce-CMEO-NPs were assessed by FT-IR spectroscopy, SEM, Zeta potential and size, and *in vitro* release of CMEO from Ce-CMEO-NPs.

#### FT-IR Analysis

The positive identification (quality) of Ce-CMEO-NPs was determined by FT-IR on a Bruker Tensor 27 spectrophotometer attached with ZnSe single crystals (Bruker, Billerica, United States) and data were analyzed using OPUS 7 spectroscopy software program. The analysis was carried out in a mode of a horizontal attenuated total reflectance (ATR) cell and working conditions were retained as relative humidity of 30% and temperature of 27°C. The samples were placed on ZnSe single crystal and infrared (IR) spectra were documented with resolution of 4 cm^-1^ at a wave number of 500–4000 cm^-1^ and baseline correction was undertaken to devoid of water vapor, CO_2_, and other background air spectrum.

#### SEM Observation

The micromorphological features of Ce-CMEO-NPs were observed under SEM as explicated in the section “SEM Observation of Macroconidia.”

#### Determination of Zeta Potential and Size

The Zeta potential and size of the synthesized Ce-CMEO-NPs were enumerated by employing Zetasizer Nano-25 (Malvern Instruments) following the methodology of Liu and Gao (2009). The Ce-CMEO-NPs were dialyzed in a 12-kDa membrane against 0.03 M Tris buffer, pH 5.6 for 18 h.

#### *In Vitro* Release of CMEO

The *in vitro* release of CMEO from Ce-CMEO-NPs was assessed by dialysis method as per the methodology of Natrajan et al. (2015). Briefly, 5 mg of Ce-CMEO-NPs was dispersed in 5 mL of phosphate buffer saline (PBS) of pH 7.4 and 0.1 M and loaded into dialysis bag. The dialysis was carried out in a beaker containing 50 mL of PBS (0.1 M) with 20% ethanol (99.99% pure) at 28°C and pH of 1.5 and 7.4 for duration of 48 h under gentle agitation. At definite time intervals (0, 6, 12, 18, 24, 30, 36, 42, and 48 h), the amount of CMEO releases from a dialysis bag was quantified spectrophotometrically (190–350 nm) using calibration curve. The CMEO release was calculated by the formula:

Release of oil (%) = Release oil/Total oil × 100

### Comparative Evaluation of Antifungal and Antimycotoxin Activity of CMEO and Ce-CMEO-NPs in Maize

The inhibitory activity of CMEO and Ce-CMEO-NPs on growth and production of DON and ZEA by *F. graminearum* in maize grains was studied under laboratory conditions. A quantity of 100 g pre-sterilized maize grains was inoculated with 10 μL spore suspension (1 × 10^6^ spores per mL) in 250 mL of Erlenmeyer flask and exposed to different concentrations of CMEO and Ce-CMEO-NPs (up to 1000 ppm) and thoroughly homogenized for 15 min at 160 rpm in a rotary shaker and incubated at 28°C for 28 days. The maize grains inoculated with fungi and not treated with either of CMEO or Ce-CMEO-NPs was referred as control. Following the incubation period, fungal growth and mycotoxins were determined by spread plate and HPLC quantification techniques, respectively.

#### Determination of Fungal Growth

The fungal growth in maize grains was determined as per our earlier described procedure of Kalagatur et al. (2018). Following incubation, maize grains (10 g) were collected from test samples and suspended in 90 mL of sterile peptone water and homogenized for 15 min at 160 rpm using a rotary shaker. Subsequently, decimal dilutions were performed and spread plated on CD agar plates and allowed to grow for 3 days at 28°C. The viability of fungi was determined in colony forming units (CFU) and expressed in log CFU/g.

#### Determination of Mycotoxins

The HPLC quantification of DON and ZEA was done as per our previously reported methodology of Kumar et al. (2016) and Mudili et al. (2014) with minor modifications. Briefly, a portion of maize grains (25 g) is milled into fine powder under aseptic conditions and suspended in 250 mL solution of acetonitrile and water (60:40, v/v) and thoroughly homogenized for 2 h at 160 rpm under a rotary shaker. The suspension was filtered through Whatman no. 1 filter paper and subjected to centrifugation at 5000 rpm for 5 min to obtain a clear supernatant. Meanwhile, immunoaffinity columns of DON and ZEA were prepared as per procedure of manufacturer, and about 20 mL of the obtained clear supernatant was passed through immunoaffinity columns at speed of three to four drops per second. The DON and ZEA were eluted in 1 mL of acetonitrile and used for HPLC quantification (Shimadzu, Japan). Briefly, analysis was executed in reverse-phase using C18 column (250 mm × 4.6 mm and 5 μm thickness) and fluorescence detector at excitation and emission wavelengths of 334 and 450 nm for ZEA, and 365 and 455 nm for DON, respectively. The solution of acetonitrile and water (50:50, v/v) was used as a mobile phase at a flow rate of 1 mL/min. The injection sample was filtered through 0.45 μm syringe filters and 25 μL was injected. The calibration curves of DON and ZEA were constructed with a different concentration and peak area, and used to deduce the quantification of mycotoxins.

### Statistical Analysis

The antifungal and antimycotoxin experiments were performed individualistically for six replicates (*n* = 6) and the results were stated as mean ± SD. The data were processed by one-way ANOVA following Tukey’s multiple comparison *post hoc* test using GraphPad Prism 7 trial version (GraphPad Software, Inc., United States). The statistical variances between the experimental sets were measured significant at *p* < 0.05.

## Results and Discussion

### Chemical Characterization of CMEO

The yield of CMEO was calculated from the dry weight of plant material employed for extraction of oil and it was found as 1.80%. The detailed chemical profile of CMEO was revealed by GC-MS analysis and a total of 46 compounds were identified constituting 95.27% (**Table 1**). The major compounds were geraniol (19.06%), geranial (14.84%), geranyl propionate (12.88%), geranyl acetone (7.35%), geranyl acetate (4.81%), α-phellandrene (2.05%), and linalool (2.01%). The results were in according to the existed reports and however, quantity of the chemical constituents varied, and this could be due to the association of different aspects, such as climate (Raina et al., 2003) part of the plant (Rajeswara Rao et al., 2009; Smitha and Rana, 2015), harvesting season (Kakaraparthi et al., 2015), nutrients (Rao, 2001), stress (Fatima et al., 1999), and extraction techniques of oil (Cannon et al., 2013).

**Table 1:** Chemical profile of *Cymbopogon martinii* essential oil (CMEO) determined by GC-MS analysis.

S. no	Compound	Retention indices	Composition (%)
1	Tricyclene	920	0.71
2	α-Pinene	933	0.30
3	Camphene	949	0.08
4	β-Pinene	977	0.06
5	6-Methyl-5-heptene-2-one	981	0.32
6	Myrcene	989	1.41
7	α-Phellandrene	1003	2.05
8	3-Carene	1010	0.53
9	Limonene	1024	1.70
10	β-Phellandrene	1027	1.10
11	(Z)-β-Ocimene	1033	1.28
12	(E)-β-Ocimene	1047	0.20
13	γ-Terpinene	1056	1.47
14	Acetophenone	1061	0.09
15	*p*-Mentha-2,4(8)-diene	1085	0.02
16	Terpinolene	1089	1.80
17	Linalool	1096	2.01
18	*n*-Undecane	1102	0.05
19	Camphor	1143	0.42
20	Borneol	1165	0.17
21	Isomenthol	1184	0.93
22	*trans*-Carveol	1217	1.50
23	Thymol	1291	5.94
24	Methyl salicylate	1294	1.23
25	Isobornyl acetate	1287	0.18
26	Bornyl acetate	1289	0.53
27	Carvacrol	1302	1.00
28	δ-Elemene	1335	0.09
29	α-Cubebene	1349	0.14
30	α-Copaene	1377	0.37
32	β-Elemene	1391	1.03
32	Geraniol	1251	19.06
33	Geranial	1263	14.84
34	Neryl acetate	1365	0.52
35	Geranyl acetate	1382	4.81
36	Geranyl acetone	1455	7.35
37	β-Santalene	1457	0.02
38	Geranyl propionate	1476	12.88
39	γ-Muurolene	1479	0.62
40	Germacrene-D	1487	1.96
41	Geraniol isobutanoate	1514	0.80
42	Elemol	1551	1.02
43	Cubenol	1642	0.38
44	Bulnesol	1671	1.14
45	Cadalene	1675	1.10
46	*cis*-Thujopsenal	1710	0.06
	Total		95.27

### Antifungal Activity

The antifungal activity of CMEO on *F. graminearum* was determined by the micro-well dilution method, and MIC and MFC values were observed at 421.7 ± 27.14 ppm and 618.3 ± 79.35 ppm, respectively. Furthermore, the antifungal mechanism of CMEO on *F. graminearum* was assessed by determining the ROS, lipid peroxidation, ergosterol, and micro-morphology of macroconidia.

**FIGURE 1:**
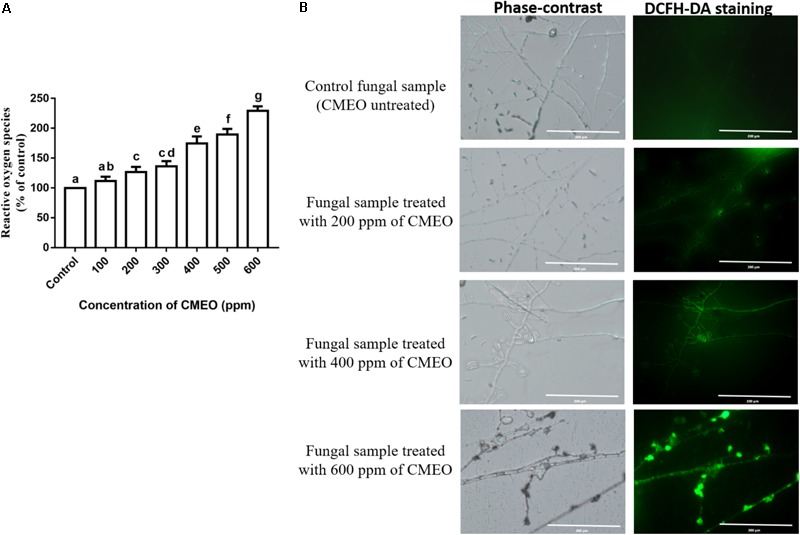
**(A)** Consequence of different concentrations of *C. martinii* essential oil (CMEO) on generation of intracellular reactive oxygen species (ROS) of *F. graminearum* determined by the dichloro-dihydro-fluorescein diacetate (DCFH-DA) staining method. Statistical data were analyzed by one-way ANOVA following Tukey’s *post hoc* multiple comparison test and columns with different letters were statistically significant (*p* < 0.05). **(B)** Phase-contrast and GFP (green fluorescent protein) images of control and CMEO-treated fungal samples. The scale bar value was 200 μm in all the images.

The ROS content in the fungal samples was determined using the DCFH-DA staining method. The hydrophobic natured non-fluorescent DCFH-DA molecule moves into the cell and hydrolyzes into DCFH molecule by intracellular esterase enzymes, and further sequential oxidation to DCF releases fluorescence molecule. The intensity of fluorescence was directly proportional to the level of ROS molecules (LeBel et al., 1992; Kumar et al., 2016). In the present study, ROS levels in CMEO-treated fungi were increased in a dose-dependent manner and results were shown in **Figure 1A**. The amount of ROS generated in CMEO-treated fungi was calculated with reference to control (100%), and it was noticed as 117.7 ± 7.24, 126.8 ± 8.40, 136.4 ± 8.53, 174.6 ± 11.65, 189.7 ± 9.36, and 229.3 ± 7.38% at 100, 200, 300, 400, 500, and 600 ppm of CMEO, respectively. In evidence, CMEO-treated fungi emitted high intensity of fluorescence compared to CMEO untreated control fungi, and fluorescence intensity was enhanced with increasing concentrations of CMEO and exhibited a dose-dependent way (**Figure 1B**). The ROS molecules (hydrogen peroxide, hydroxyl, and peroxide radicals) play an important role in the biological system, and enhanced level of intracellular ROS molecules destructs cellular components like DNA, RNA, protein, and lipids and even damages the integrity of cellular membrane. This process triggers oxidative stress-mediated apoptotic death of fungi by inhibition of spore germination and biomass production (Liu et al., 2010) and this could be one of the major reasons for antifungal activity of CMEO on *F. graminearum*.

**FIGURE 2:**
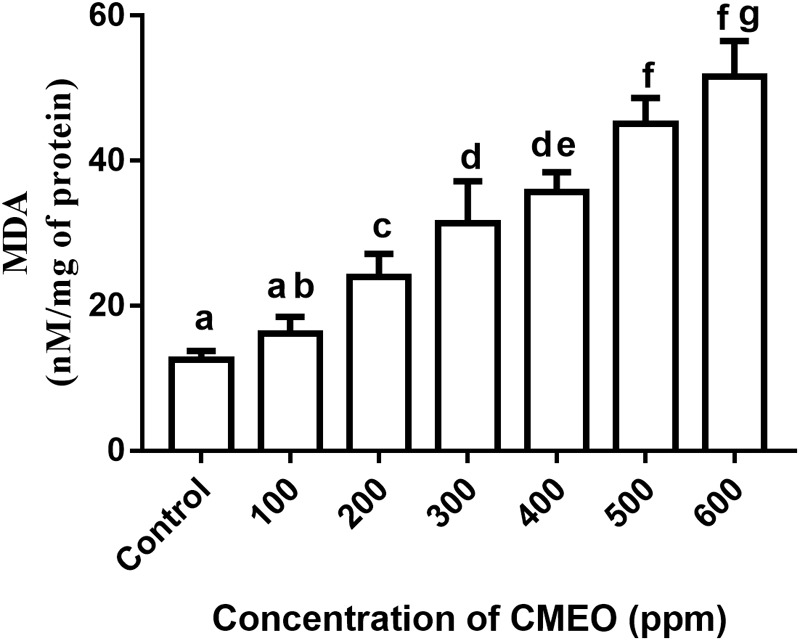
Effect of different concentrations of *C. martinii* essential oil (CMEO) on lipid peroxidation of *F. graminearum*. Statistical data were analyzed by one-way ANOVA following Tukey’s *post hoc* multiple comparison test and columns with different letters were statistically significant (*p* < 0.05).

Furthermore, the effect of CMEO on lipid peroxidation of fungi was assessed by determining the levels of MDA. The methylene –CH_2_– groups of polyunsaturated fatty acids are adversely affected by ROS, and thereby promotes peroxidation of lipids. The lipid peroxidation disturbs the integrity and function of the membrane integrity and produces the aldehyde by-product MDA, which is referred as an indicator for lipid peroxidation. The MDA reacts with DNA and form propane adduct with 2’-deoxyguanosine (M1G-dR), which extremely influence various physiological functions of cell, including cell signaling, cell proliferation and differentiation, and apoptosis (Vasilaki and McMillan, 2011). In the present study, MDA levels in fungi were enhanced with treatment of CMEO compared to control (**Figure 2**). It was determined as 12.58 ± 1.17, 16.12 ± 2.36, 23.96 ± 3.20, 31.43 ± 5.73, 35.72 ± 2.72, 45.12 ± 3.53, and 51.59 ± 4.94 nM/mg of protein at 0 (control), 100, 200, 300, 400, 500, and 600 ppm of CMEO, respectively. The accomplished MDA levels agreed with results of ROS analysis, and the increase in MDA levels could be due to the detrimental interaction of ROS with lipids. The study concluded that antifungal activity of CMEO could be through the detrimental effect on cellular membrane integrity by generation of ROS molecules and lipid peroxidation. In support of our study, Tian et al. (2012) and Gao et al. (2016) have evidently demonstrated that essential oils and its active constituents detrimentally affect the plasma membrane and mitochondria and activate apoptotic death of fungi by elevating intracellular ROS and lipid peroxidation.

**FIGURE 3:**
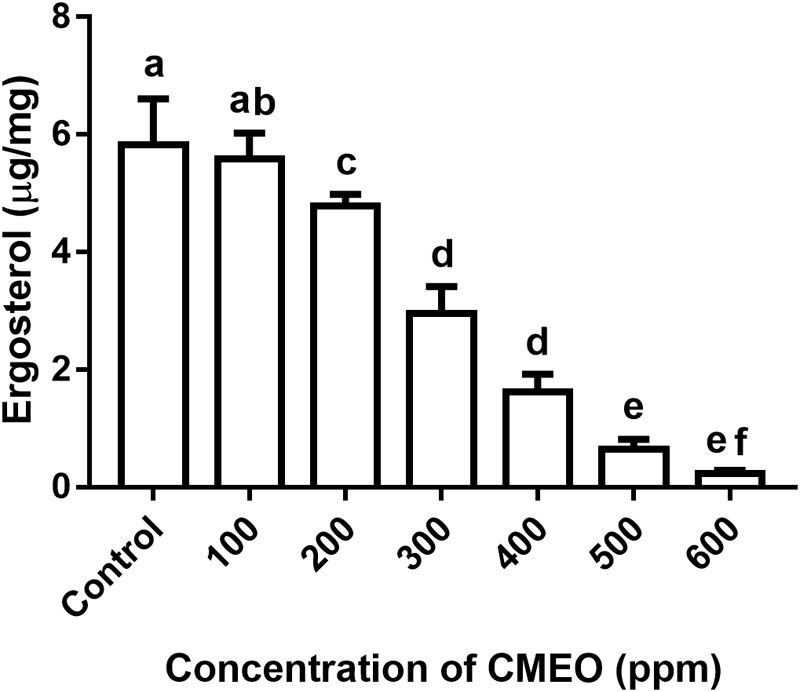
Effect of different concentrations of *C. martinii* essential oil (CMEO) on ergosterol content of *F. graminearum*. Statistical data were analyzed by one-way ANOVA following Tukey’s *post hoc* multiple comparison test and columns with different letters were statistically significant (*p* < 0.05).

On the other hand, ergosterol content was relatively less in CMEO-treated fungi compared to control and it was exhibited a dose-dependent fashion of reduction (**Figure 3**). A quantity of 5.83 μg/mg of ergosterol was noticed in control fungal sample. While the quantity of ergosterol in CMEO-treated fungal samples was determined as 5.58 ± 0.43, 4.78 ± 0.20, 2.95 ± 0.46, 1.62 ± 0.30, 0.64 ± 0.16, and 0.23 ± 0.05 μg/mg at 100, 200, 300, 400, 500, and 600 ppm, respectively. The lipid ergosterol is exclusively found in cellular membranes of fungi and protozoa, and it was crucial for maintaining the integrity and fluidity of cellular membrane similar to cholesterol of animal cells. Ergosterol is measured as one of the indicators for living fungal biomass and inhibition of ergosterol biosynthesis is a major target site for most of the antifungal drugs like amphotericin B, miconazole, itraconazole, and clotrimazole. Hence, inhibition of ergosterol synthesis was one of the foremost targets to discovery of new antifungal agents (Mille-Lindblom et al., 2004; Sellamani et al., 2016). In the present study, CMEO has successfully decreased the ergosterol levels in fungi and this could be one of vital reasons for antifungal activity.

**FIGURE 4:**
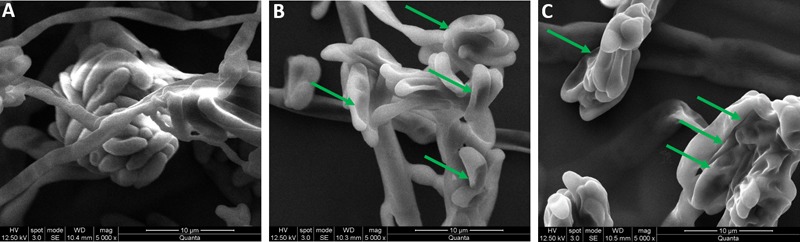
Micro-morphological feature of fungal macroconidia under SEM. **(A)** Control macroconidia. Panels **(B,C)** were macroconidia treated with minimum inhibitory concentration (MIC) and minimum fungicidal concentration (MFC) of *C. martinii* essential oil (CMEO), respectively. Arrows shows the detrimental micro- morphological features.

In conclusion, the antifungal effect of CMEO on *F. graminearum* was confirmed by observing the micro-morphology of macroconidia by SEM at MIC and MFC values. The CMEO-treated macroconidia were exhibited some distinctive morphological features compared to control (**Figure 4**). The control macroconidia were healthy with homogenous, turgid, and smooth surface (**Figure 4A**). While CMEO-treated macroconidia showed detrimental micro-morphological features such as squashed, rough surface, collapsed, dispersed, wrinkled, protuberances, craters, and vesicles (**Figure 4**). Interestingly, severe detrimental micro-morphological changes in macroconidia were perceived at MFC compared to MIC value of CMEO (**Figures 4B,C**). The study concluded that antifungal activity of CMEO could be due to the detrimental impairment of micro-morphology of macroconidia, and it could be due to the upsurge of ROS and MDA levels, and exhaustion of membrane integrity and ergosterol content.

The potent antifungal activity of CMEO was owed to its chemical constituents such as geraniol, geranial, linalool, thymol, limonene, α-phellandrene, ocimene, germacrene-D, and isomenthol. These aromatic compounds cause permeabilization of cellular membranes by destructing the lipids and proteins, and brings coagulation of cytoplasm, leakage of ions, breakdown of proton pump, drop in mitochondrial membrane potential and reduction of ATP molecules, release of cytochrome c and these concurrent events boost the death of fungi by apoptosis (Prashar et al., 2003; Bakkali et al., 2008; Ramsdale, 2008; Sharon et al., 2009; Gao et al., 2016). Furthermore, Food and Drug Administration (FDA) of United States has recognized CMEO as GRAS considering the safety, eco-friendly, non-toxic, antioxidant, and antimicrobial reports (Burt, 2004). Additionally, Kumar et al. (2007) have noticed insect repellent properties of CMEO in cereals and legumes and recommended to use as an insecticide in agricultural commodities. In addition to these multi-beneficial features, the present study demonstrated the antifungal activity of CMEO on post-harvest plant pathogen *F. graminearum* and recommended as mycobiocide in agriculture and food industry.

### Characterization of Ce-CMEO-NPs Nanoparticles

The conventional agriculture is quite often characterized by excess application of synthetic mycobiocides, which generally cause environmental pollution, health issues in humans and animals, and develop fungicide-resistant fungi (Lucas et al., 2015). Therefore, researchers have given wide attention for discovery of alternatives to hazardous synthetic mycobiocides (George et al., 2016). Several researchers have found that plant-based mycobiocides are better alternatives for synthetic mycobiocides and especially, essential oils are non-toxic, biodegradable, “GRAS” and appropriate for sustainable agriculture (Burt, 2004). However, essential oils are highly volatile and easily degrade on exposure to light, heat, pH, moisture, and oxygen, and thus, deprive their stability and bio-functional properties. To overcome instability of essential oils, nanotechnologists have developed the technique of microencapsulation of essential oils with chitosan, alginates, zein protein, etc. Thus, microencapsulation safeguards the essential oils from degradation and enables its perseverance, and monitored and gradual release (Turasan et al., 2015; Bakry et al., 2016; Malešević, 2016).

**FIGURE 5:**
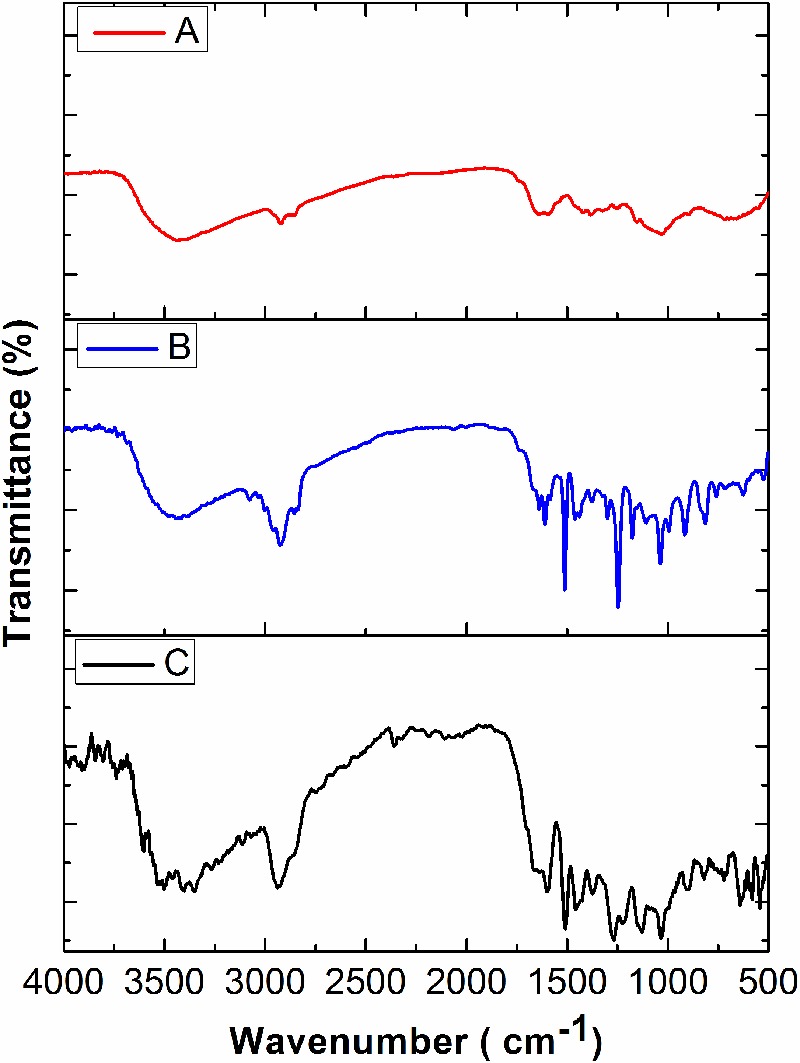
Fourier transform infrared (FT-IR) spectra of **(A)** chitosan, **(B)** *C. martinii* essential oil (CMEO), and **(C)** chitosan encapsulated *C. martinii* essential oil nanoparticles (Ce-CMEO-NPs).

**FIGURE 6:**
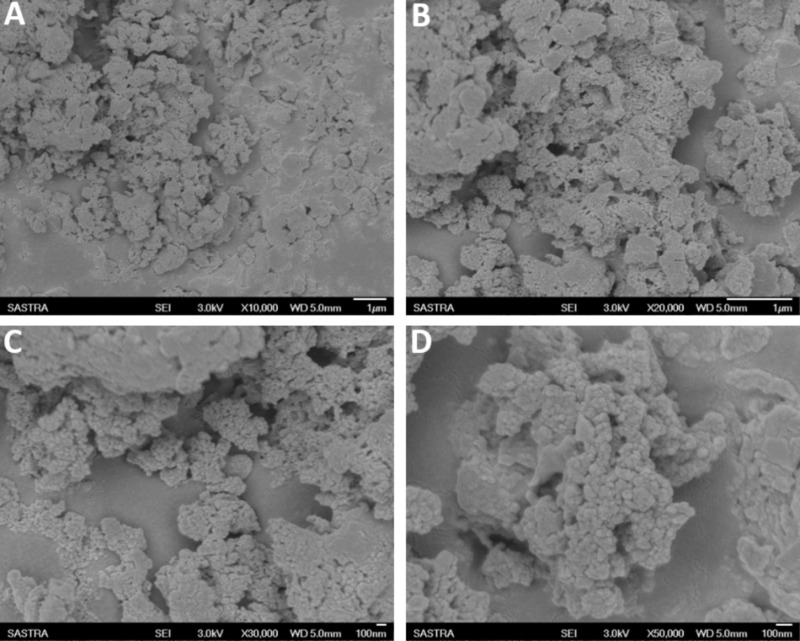
Scanning electron microscopic (SEM) image of chitosan encapsulated *C. martinii* essential oil nanoparticles (Ce-CMEO-NPs) loaded with 700 ppm of *C. martinii* essential oil (CMEO) at different magnifications of **(A)** 10,000×, **(B)** 20,000×, **(C)** 30,000×, and **(D)** 50,000×.

In the present study, various concentrations of CMEO were successfully encapsulated with chitosan by the emulsification process (Ribeiro et al., 2013). The obtained Ce-CMEO-NPs were characterized by FT-IR, SEM, Zeta potential, and size analysis. The *in vitro* release of CMEO from Ce-CMEO-NPs was also investigated in detail. The FT-IR spectra of the (A) chitosan, (B) CMEO, and (C) Ce-CMEO-NPs were detailed in **Figure 5** and revealed the successful encapsulation of essential oil with chitosan. The results depict the characteristic absorption bands of chitosan, CEMO, and Ce-CMEO-NPs. The band attendance at 3427 cm^-1^ in chitosan, CMEO, and Ce-CMEO-NPs represents O-H vibration, which confirms one of the positive signs for encapsulation of chitosan with CMEO. Furthermore, peaks at 2935, 1407, 1038, and 1607 cm^-1^ in CMEO and Ce-CMEO-NPs represent the stretching vibrations of C–H, C–N, C–O–C, and N–H, respectively (Gayathri et al., 2015). These results signify the successful encapsulation of Ce-CMEO-NPs (**Figures 5B,C**). The existence of amine groups on the surface of the chitosan is measured as the key to bind to the active functional groups of CMEO. Especially, unique peak at 1607 cm^-1^ is due to the presence of amine group in chitosan, which is considered to be pivotal in bioconjugation and formation of Ce-CMEO-NPs (**Figures 5B,C**). The amino group complexes are having unoccupied coordination points, which act as the binding sites for functional ligands of CMEO constituent molecules like geraniol, geranial, limonene, linalool, and limonene. The bio-active molecules in CMEO are more stabilized due to chitosan conjugation and enhance the effectiveness of the antifungal activity. The morphological feature of Ce-CMEO-NPs was observed by SEM and depicted in **Figure 6**. The microscopic images revealed that the Ce-CMEO-NPs were showing spherical surface morphology. This is because of high thermodynamic and shape stability of Ce-CMEO-NPs. The agglomerated surface morphology of the prepared Ce-CMEO-NPs is due to the electrostatic interaction of chitosan fragments, which are branched biopolymer.

Furthermore, stability and size of Ce-CMEO-NPs were determined by Zeta potential and size distribution analysis. We have prepared Ce-CMEO-NPs with various loading concentrations of CMEO (ppm). The obtained Zeta potential values are ranging from 39.3 to 37.2 mV for the corresponding particle size of Ce-CMEO-NPs varying from 455 to 480 nm (**Table 2**). The obtained Zeta potential values above 35 mV confirm that the Ce-CMEO-NPs are highly stable, and the particle size of the Ce-CMEO-NPs is not greatly affected by the CMEO loading.

**Table 2:** Zeta potential and size of chitosan encapsulated CMEO nanoparticles (Ce-CMEO-NPs) made with different ppm of CMEO.

	100 ppm	200 ppm	300 ppm	400 ppm	500 ppm	600 ppm	700 ppm
Particle size (nm)	455	459	464	468	472	476	480
Zeta potential (mV)	39.3	38.9	38.3	38.1	37.8	37.6	37.2

**FIGURE 7:**
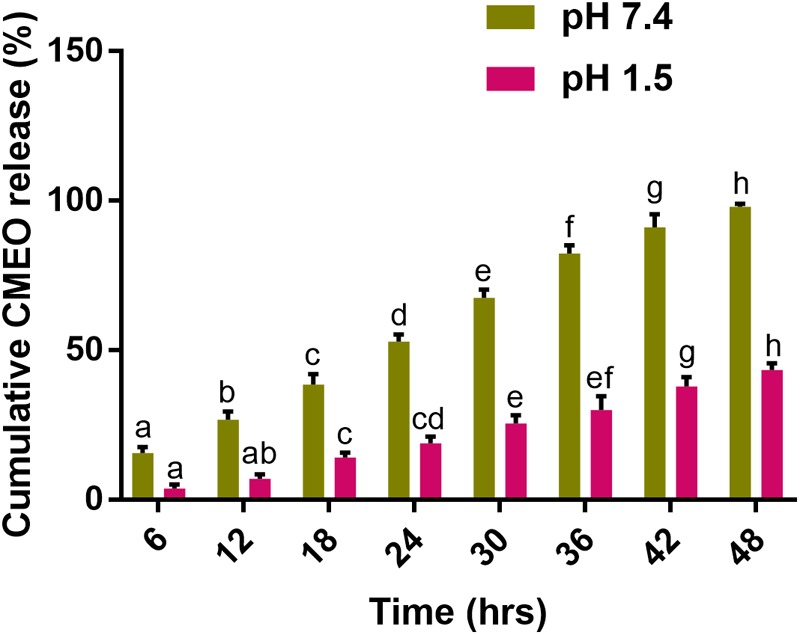
*In vitro* release kinetics of *C. martinii* essential oil (CMEO) from chitosan encapsulated *C. martinii* essential oil nanoparticles (Ce-CMEO-NPs) in hydroethanolic solution at pH 7.4 and 1.5. Statistical data were analyzed by one-way ANOVA following Tukey’s *post hoc* multiple comparison test and columns with different letters were statistically significant within the group of pH 7.4 and 1.5 (*p* < 0.05).

The *in vitro* cumulative release of CMEO from Ce-CMEO-NPs at pH 7.4 and 1.5 over a period of 48 h is shown in **Figure 7**. It was noticed that the better-sustained release of CMEO at pH 7.4 than pH 1.5. For a period of 48 h, cumulative of 97.94 ± 0.95 and 43.37 ± 2.17% of CMEO was released from Ce-CMEO-NPs at pH 7.4 and pH 1.5, respectively. The reason might be the formation of highly stable bioactive complex of chitosan at acidic pH 1.5 (Cojocariu et al., 2012). It is observed that the release of essential oil is stabilized due to the complex formation with chitosan. The volatile alcohols and phenol groups of CMEO will be converted into its more stable complex forms by the chitosan encapsulation process which in turn increases their effective lifetime for fungicidal action by gradual release of antifungal constituents.

### Comparative Evaluation of Antifungal and Antimycotoxin Activities of CMEO and Ce-CMEO-NPs in Maize

**FIGURE 8:**
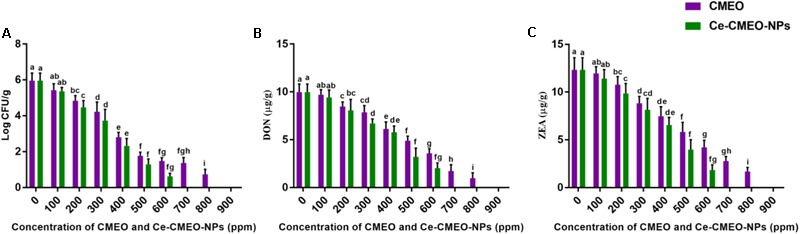
*In vitro* inhibitory effect of different concentrations (ppm) of *C. martinii* essential oil (CMEO) and chitosan encapsulated *C. martinii* essential oil nanoparticles (Ce-CMEO-NPs) on **(A)** growth, and level of **(B)** deoxynivalenol (DON) and **(C)** zearalenone (ZEA) by *F. graminearum* in maize. Statistical data were analyzed by one-way ANOVA following Tukey’s *post hoc* multiple comparison test and columns with different letters were statistically significant within the group of CMEO and Ce-CMEO-NPs (*p* < 0.05).

**FIGURE 9:**
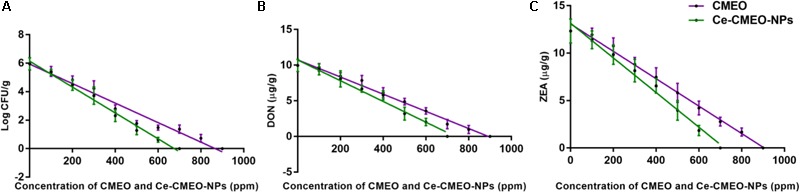
Linear regression curves for inhibitory effect of different concentrations (ppm) of *C. martinii* essential oil (CMEO) and chitosan encapsulated *C. martinii* essential oil nanoparticles (Ce-CMEO-NPs) on **(A)** growth, and level of **(B)** deoxynivalenol (DON) and **(C)** zearalenone (ZEA) by *F. graminearum* in maize.

Antifungal and antimycotoxin activities of CMEO and Ce-CMEO-NPs on *F. graminearum* in maize grains were conducted under laboratory conditions over a storage period of 28 days. The CMEO and Ce-CMEO-NPs have presented potent fungicidal and antimycotoxin activities in maize grains (**Figure 8**). The kinetic curves for reductions of fungal growth and level of mycotoxins (DON and ZEA) with different concentrations of CMEO and Ce-CMEO-NPs were depicted in **Figure 9**. The constructed regression models were found satisfactory and presented a good determination coefficient (*R*^2^) of 0.9694 and 0.9896 (log CFU), 0.9864 and 0.9793 (DON), and 0.9935 and 0.9873 (ZEA) for CMEO and Ce-CMEO-NPs, respectively (Supplementary Table 1). These regression models conveyed that reductions of fungal growth and mycotoxins were dependent on the concentration of CMEO and Ce-CMEO-NPs and show a dose-dependent fashion. Though, Ce-CMEO-NPs have exhibited potent antifungal and antimycotoxin activities compared to CMEO (**Figure 8**). The complete reductions of fungal growth and mycotoxins were noticed at 700 ppm of Ce-CMEO-NPs and 900 ppm of CMEO (**Figure 8**). The essential oils are volatile and susceptible to degradation and loss of bio-functional features. Whereas essential oils encapsulated in chitosan are less prone to degradation, perseverance bio-functional properties, and gradually releases the bio-active elements in a controlled way (Turasan et al., 2015; Bakry et al., 2016; Malešević, 2016). Consequently, Ce-CMEO-NPs are highly resistant to degradation, perseverance bio-functional features, and slowly release active antifungal constituents compared to CMEO. Henceforth, Ce-CMEO-NPs have exhibited potent antifungal and antimycotoxin activities compared to CMEO. In support of the present study, Khalili et al. (2015) have established the greater antifungal activity of chitosan encapsulated thyme essential oil nanoparticles over thyme essential oil during safeguarding the tomato fruit against *A. flavus*. Similarly, Zhaveh et al. (2015) have also proven the enhanced antifungal activity of chitosan-caffeic acid encapsulated *Cuminum cyminum* essential oils nanogels related to *C. cyminum* essential oils against *A. flavus*. The findings of the present study concluded that Ce-CMEO-NPs were highly effective related to CMEO in safeguarding the stored grains from fungi and its hazardous mycotoxins.

## Conclusion

CMEO was extracted by hydrodistillation technique and chemical profile was unveiled by GC-MS analysis. A total of 46 chemical constituents were identified and major compounds were geraniol, geranial, geranyl propionate, geranyl acetone, geranyl acetate, α-phellandrene, linalool, and thymol. The CMEO was induced the death of *F. graminearum* through elevating ROS generation and lipid peroxidation, depleting ergosterol content, and detrimentally affecting the micromorphology of macroconidia. Furthermore, Ce-CMEO-NPs were successfully synthesized with the diameter of 100 nm and spherical shape. The FT-IR analysis concluded that chitosan was successfully enclosed the essential oil. The *in vitro* essential oil release assay concluded the persistence of essential oil with a slow and controlled release. In the final aim of study, antifungal and antimycotoxin activities of CMEO and Ce-CMEO-NPs against *F. graminearum* were established in maize grains under laboratory conditions over a storage period of 28 days. The study determined that Ce-CMEO-NPs have potent antifungal and antimycotoxin activities compared to CMEO due to perseverance of antifungal activity by the controlled release of fungicidal constituents. The Ce-CMEO-NPs could be highly applicable as mycobiocide in safeguarding the agriculture commodities from mycotoxigenic fungi.

## Author Contributions

NK and VM were designed the work. NK, NS, ONG, and VM were performed the experiments, analyzed the data, and drafted the manuscript. All authors approved the final version of the manuscript.

## Conflict of Interest Statement

The authors declare that the research was conducted in the absence of any commercial or financial relationships that could be construed as a potential conflict of interest.
