# Supplementary material for: Antifungal Activity of Chitosan Nanoparticles Encapsulated With Cymbopogon martinii Essential Oil on Plant Pathogenic Fungi Fusarium graminearum
Source: Front Pharmacol. 2018 Jun 6;9:610. doi: 10.3389/fphar.2018.00610 (PMC5997812; doi:10.3389/fphar.2018.00610)
Supplement: Supplementary file 1 [file Table_1.DOCX]

**Supplementary Table 1:** Linear regression curve fit for inhibitory effect of *C. martinii* essential oil (CMEO) and chitosan encapsulated *C. martinii* essential oil nanoparticles (Ce-CMEO-NPs) on growth (log CFU), deoxynivalenol (DON), and zearalenone (ZEN) by *F. graminearum* in maize.

|  |  | Log CFU/g | |  | DON (µg/g) | |  | ZEA (µg/g) | |
| --- | --- | --- | --- | --- | --- | --- | --- | --- | --- |
| Best-fit values ± SE |  | CMEO | Ce-CMEO-NPs |  | CMEO | Ce-CMEO-NPs |  | CMEO | Ce-CMEO-NPs |
|  |  |  |  |  |  |  |  |  |  |
| Slope |  | -0.006844 ± 0.0004297 | -0.009085 ± 0.0003795 |  | -0.01203 ± 0.0004986 | -0.01454 ± 0.0008627 |  | -0.01444 ± 0.0004129 | -0.01825 ± 0.000846 |
| Y-intercept |  | 5.937 ± 0.2294 | 6.143 ± 0.1588 |  | 10.74 ± 0.2662 | 10.73 ± 0.3609 |  | 13.08 ± 0.2204 | 13.15 ± 0.3539 |
| X-intercept |  | 867.4 | 676.2 |  | 892.8 | 737.9 |  | 906 | 720.5 |
| 1/slope |  | -146.1 | -110.1 |  | -83.12 | -68.77 |  | -69.26 | -54.78 |
| 95% confidence intervals |  |  |  |  |  |  |  |  |  |
| Slope |  | -0.007835 to -0.005853 | -0.01001 to -0.008156 |  | -0.01318 to -0.01088 | -0.01665 to -0.01243 |  | -0.01539 to -0.01349 | -0.02032 to -0.01618 |
| Y-intercept |  | 5.408 to 6.466 | 5.755 to 6.532 |  | 10.13 to 11.35 | 9.846 to 11.61 |  | 12.57 to 13.59 | 12.29 to 14.02 |
| X-intercept |  | 801.7 to 951.1 | 638.6 to 720.8 |  | 846 to 947.7 | 679.6 to 812.9 |  | 872.3 to 943.7 | 675.3 to 775.2 |
| Goodness of fit |  |  |  |  |  |  |  |  |  |
| R square |  | 0.9694 | 0.9896 |  | 0.9864 | 0.9793 |  | 0.9935 | 0.9873 |
| Sy.x |  | 0.3903 | 0.246 |  | 0.4529 | 0.5591 |  | 0.375 | 0.5483 |
| Is slope significantly non-zero? |  |  |  |  |  |  |  |  |  |
| F |  | 253.7 | 572.9 |  | 582.2 | 284.1 |  | 1223 | 465.6 |
| DFn, DFd |  | 1, 8 | 1, 6 |  | 1, 8 | 1, 6 |  | 1, 8 | 1, 6 |
| *P* value |  | < 0.005 | < 0.005 |  | < 0.005 | < 0.005 |  | < 0.005 | < 0.005 |
| Deviation from zero? |  | Significant | Significant |  | Significant | Significant |  | Significant | Significant |
| Equation |  | Y = -0.006844*X + 5.937 | Y = -0.009085*X + 6.143 |  | Y = -0.01203*X + 10.74 | Y = -0.01454*X + 10.73 |  | Y = -0.01444*X + 13.08 | Y = -0.01825*X + 13.15 |
